# A Structured Approach to Skin and Soft Tissue Infections (SSTIs) in an Ambulatory Setting

**DOI:** 10.3390/clinpract11010011

**Published:** 2021-02-01

**Authors:** Benjamin Silverberg

**Affiliations:** Department of Emergency Medicine, West Virginia University, 1 Medical Center Drive, Box 9149, Morgantown, WV 26506, USA; benjamin.silverberg@hsc.wvu.edu

**Keywords:** inflammation, purulence, drainage, necrosis, MRSA, immunodeficiency, trauma, antibiotics, resistance

## Abstract

The skin is the largest, and arguably, the most vulnerable organ in the human body. Scratches and scrapes, bites and puncture wounds, impetigo and erysipelas—all these disruptions can lead to pain, swelling, and/or systemic symptoms. In this article, which is based on the Infectious Diseases Society of America’s 2014 guidelines and the World Society of Emergency Surgery and Surgical Infection Society of Europe’s 2018 consensus statement, a structured approach to skin and soft tissue infections (SSTIs) is reviewed, comparing treatment for suppurative and non-suppurative infections, and then discussing specific conditions commonly seen in Primary Care and Urgent Care facilities.

## 1. Introduction

Skin and soft tissue infections (SSTIs) result from a compromise of the skin’s defenses and microbial invasion and interaction therein. Since SSTIs are usually caused by bacteria, most practice guidelines do not mention viral, fungal, or parasitic etiologies. Trauma and surgery are two of the main ways the skin barrier can be breached [1]. Primary SSTIs result from the invasion of otherwise healthy skin; secondary SSTIs result from infection of already-damaged skin, such as from trauma or an underlying disease [2]. Infections are often localized but they can also spread via the blood stream or lymphatic flow [3].

## 2. Background

In the United States, SSTIs account for more than 14 million outpatient visits annually [3,4]. About three-fourths of cases of SSTIs are managed in an outpatient setting. However, since mild infections are often self-limiting, many patients do not seek formal medical care [2,5]. As such, these numbers are likely underestimates of the true incidence. SSTIs are a common reason for a trip to the Emergency Department, with 59% of infections being attributed to methicillin-resistant *Staphylococcus aureus* (MRSA) [3,6] and 11.5–60% of patients being admitted to the hospital for an expensive multi-day stay [6]. Men are disproportionately affected: They account for 60–70% of SSTIs [5].

## 3. Discussion

The skin—the largest organ in the body—protects the occupant with a physical barrier, bacteriostatic sebaceous fluid, and normal skin flora [5]. This normal bacterial colonization is mainly with aerobic Gram-positive cocci, but below the waist, due to a so-called “fecal veneer” stemming from the anorectal region, Gram-negative species can also be found [5]. Additionally, the warm, dark, moist regions in skin folds tend to have higher concentrations of bacteria [5]. In undamaged skin, microflora tend to reside in the outer layers of the skin and skin structures. Anaerobic bacteria tend to be associated with long-standing skin ulcers, impaired immune function, and recent antibiotic use [5].

Aerobic Gram-positive cocci—specifically *Staphylococcus aureus* and streptococcal species—are the most likely cause of SSTIs involving healthy skin. Beta-hemolytic streptococci cause nearly three-fourths of cases of cellulitis [3,7]. *S. aureus*, by comparison, tends to cause more purulent infections, such as abscesses [2]. There is a considerable regional variation: Nearly 36% of *S. aureus* colonies in North America display methicillin resistance, compared to 29.4% in Latin America and 22.8% in Europe [1]. Though simple infections are usually monomicrobial, complicated infections can be polymicrobial or monomicrobial, as well [2].

### 3.1. Conceptual Frameworks for SSTIs

SSTIs were originally categorized by the US Food and Drug Administration (FDA) in 1998 as “uncomplicated” or “complicated,” mainly for the purpose of therapeutic drug studies [8,9]. Though these definitions would later be revised, certain types of wounds (e.g., diabetic ulcers) were excluded. In 2003, Eron et al. proposed a new way to stratify SSTIs by severity. Their four-tier system ranged from a healthy-appearing patient through a febrile and ill-appearing one to an overtly septic patient with a life-threatening infection [1,3,5,10,11]. Criticizing it as ambiguous, Ki and Rotstein (2008) tried to improve on this system by adding in objective criteria such as comorbidities, systemic signs, involvement of the head and/or hands, and involvement of more than 9% of the body surface area [5,8]. Various iterations of early warning scores (EWS), which focus primarily on vital signs, have also been used for hospitalized patients [8,11].

**Figure 1:**
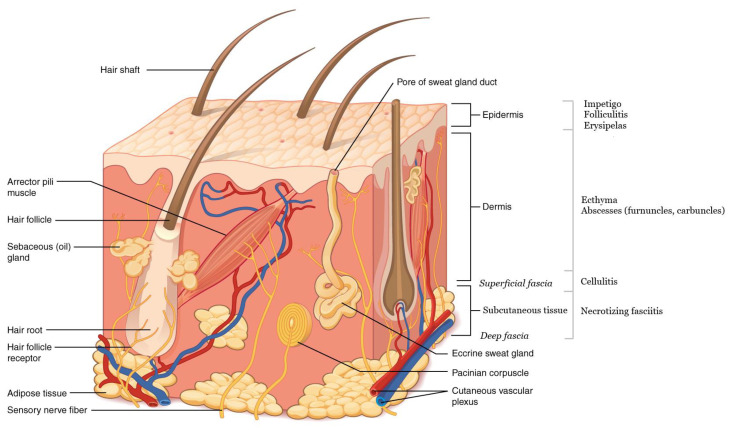
Infectious processes by depth. Soft tissue structures and layers of the skin are also marked (adapted from Figure 5.2 of Anatomy and Physiology, 2013, via OpenStax College; licensed through Creative Commons–https://commons.wikimedia.org/wiki/File:501_Structure_of_the_skin.jpg).

Alternatively, SSTIs can be grouped by the anatomic tissue layers involved, as seen in Figure 1 [1,12,13]. Cellulitis is typically in the dermis and subcutaneous tissue, and necrotizing infections most commonly affect the deep fascia, but can involve the dermis down through to the muscle [1,4,9].

A third way to classify SSTIs is whether they are suppurative (pus-forming) or not [1,3,14]. Localized purulent staphylococcal infections result in the formation of furuncles (boils), carbuncles (clusters of boils), or larger skin abscesses. Erysipelas, cellulitis, and necrotizing fasciitis are usually non-purulent, but not always [2].

### 3.2. History of Present Illness (HPI)

There are often clues in the patient’s history suggesting the depth and severity of the SSTI. Such indicators include: Was there a traumatic injury? If so, how did it damage the skin’s defenses? Does the patient have any comorbidities? Diabetes mellitus, a risk factor for both MRSA and necrotizing fasciitis (NF), can affect a person’s peripheral sensation and wound healing. Venous stasis also affects the oxygenation of tissue, and edema can stretch and strain the skin itself. Surgery can impair the lymph flow. Immunocompromise—due to an underlying condition or medical treatment—further weakens the host’s defenses. Table 1 lists these and other risk factors for SSTIs [1,3,4,5,10,12].

### 3.3. Physical Exam

SSTIs are usually heralded by the signs of inflammatory response—erythema, warmth, tenderness/pain, and swelling—and other localized and systemic manifestations [5]. (Fluor, or secretion, has been proposed as a fifth sign of inflammation, but funcio laesa, or loss of function, is the most commonly added element to this framework. Loss of function results most directly from pain and swelling). A patient presenting with just skin redness, for example, is more likely to have a superficial, and therefore mild, infection. Systemic signs such as fever, hypotension, and tachycardia suggest a deeper infection. Physical findings such as fluctuance, bullae, crepitus, purpura, and/or necrosis suggest a more serious infection and/or one needing surgical intervention [10]. The rapid progression of symptoms, lymphangitic spread, and, in particular, pain out of proportion to exam, which may suggest tissue ischemia, are also worrisome [3,4]. Staphylococcal toxic shock syndrome (TTS), for example, which may appear to be more superficial in terms of skin depth, is accompanied by signs of shock [15]. Therefore, the entirety of the patient’s presentation must be considered when ascertaining severity.

### 3.4. Imaging

Plain radiographs have limited utility in the diagnosis or treatment of simple SSTIs in an outpatient clinic [3]. Ultrasound can be used to evaluate abscesses and fascial inflammation, which may be suggested by fluctuance or crepitus on physical exam [5]. MRI, which is more sensitive, and CT can also image fascial planes, but they typically would not change outpatient management. In more serious cases, surgical intervention—if needed—should not be delayed for imaging [4,16].

### 3.5. Laboratory Studies

Simple, localized SSTIs typically do not need laboratory evaluation [4]. However, obtaining a complete blood count, basic metabolic profile, and C-reactive protein may be appropriate if the patient is likely to be transferred to a higher-level care. Wound cultures have a somewhat low yield, and it can be challenging sometimes to distinguish between pathogenic bacteria and normal skin colonization. Blood cultures also have a low yield, unless risk factors are present. Cultures may be more useful in patients who are hospitalized and febrile, and/or with an underlying disease [4,14,17,18,19,20,21].

### 3.6. Principles of Treatment

It has been difficult to standardize the classification and, thereby treatment, of SSTIs. Antimicrobials are typically initiated before a specific etiology is confirmed. Therefore, the selection of an appropriate regimen is usually empiric, based on the bodily location and perceived severity of the infection. There is a higher perceived risk of loss of function with infections involving the hands or head, for instance, so the treatment of infections there should be aggressive. For SSTIs below the waist, pharmacotherapy should cover Gram-negative and anaerobic bacteria. *Pseudomonas aeruginosa* is possible with a chronic, non-healing ulcer [5].

As noted earlier, purulence—or absence thereof—is one fairly easy way to narrow the differential diagnosis and hone the treatment plan. In each of these groupings, assess whether the infection is mild, moderate, or severe. Though there is a debate in the literature as to whether IV/IM antibiotics are truly superior to PO antibiotics in terms of efficacy (parenteral antibiotics at least seem to deliver higher levels of the drug more rapidly, though it is unclear if outcomes are any better), the general consensus is to use PO antibiotics for mild infections and IV/IM ones for severe infections [5]. Table 2 summarizes treatment modalities.

The treatment for SSTIs attributed to MSSA or MRSA typically lasts 7–14 days, but it should be individualized based on the patient’s clinical response [5,8,14]. Similarly, reevaluate the diagnosis and treatment plan if the patient fails to show improvement after five days’ worth of antibiotics [5]. An appropriately-treated SSTI usually shows a reduction in inflammation and no further spreading within 48–72 h of antibiotic administration [2,4]. Clindamycin is an important therapeutic option to treat MSSA and MRSA in children [6], but there is increasing resistance to the drug [1]. Trimethoprim-sulfamethoxazole, doxycycline, and minocycline also treat both MSSA and MRSA [1,14]. Local resistance patterns, cost of treatment, ease of adhering to the dosing schedule, and likelihood and tolerability of side effects are things to consider when selecting one antibiotic over another [3].

### 3.7. Hospitalization

Indications for hospitalization include intolerance of oral antibiotics or apparent progression of the infection despite the outpatient treatment. Signs portending a bad outcome—tissue necrosis, hypotension, severe pain, altered mental status, and organ failure, for example—also warrant in-patient management with broad-spectrum coverage until specific sensitivities are available. The need for surgical intervention under anesthesia, as with deep or hard-to-access infections, is yet another reason for hospitalization [3].

### 3.8. Adjuvant Therapies

Oral steroids could potentially be used as an adjunct to antibiotics in non-diabetic adults with severe cellulitis, but more research is needed [2,10,14]. If they are available, negative-pressure wound therapy (NPWT) devices seem to promote wound healing after a surgical site infection (SSI), diabetic wound infection, or burn [1,22], though there is some debate on the quality of the available research [23]. Hyperbaric oxygen therapy (HBO) may be useful in some situations (e.g., necrotizing infections), but not if it delays the current standard of care. More evidence is needed [1,9,10,14].

## 4. Diagnosis and Treatment

### 4.1. Impetigo

Impetigo, a superficial, non-purulent SSTI, is characterized by an itchy, vesicular rash on the face or extremities that evolves into pustules and, subsequently, golden, honey-colored crusts. Since this SSTI is usually caused by streptococcus species, acute rheumatic fever or glomerulonephritis are possible complications. For localized, non-bullous lesions, prescribing guidelines in the United Kingdom advise the hydrogen peroxide cream [24]. Per the Infectious Diseases Society of America (IDSA), though, a 5-day course of topical mupirocin or retapamulin ointment is the first-line therapy [14]. Oral antibiotics active against MSSA may be needed if the infection is not improving within 3–5 days of initial treatment, though some authors suggest it may take up to 7 days to see an improvement [14]. Warm water soaks can help remove the crusts, but take care to avoid further insult to the skin’s integrity [2].

### 4.2. Ecthyma

Ecthyma is a deeper form of impetigo also known as ulcerative pyoderma. This should not be confused with the autoimmune condition pyoderma gangrenosum. Ecthyma features crusted sores with ulcers beneath them, usually extending into the dermis. If there is pus, the sores usually prevent easy drainage or successful treatment with topical antibiotics. Therefore, oral antibiotics for 7 or more days are indicated [14]. A gentle debridement of the crusts may be helpful, but it may be best to defer this to a Dermatologist or Infectious Disease specialist.

### 4.3. Folliculitis

Acute bacterial folliculitis involves the infection of one or more hair follicles. The most common form of superficial folliculitis has the somewhat confusing eponym “impetigo of Bockhart” and is caused by *S. aureus*. Recurrent folliculitis is usually due to community-acquired MRSA. Deeper folliculitis may be chronic and associated with shaving hair-bearing areas. “Hot tub” folliculitis is associated with *P. aeruginosa*. The differential diagnosis also includes viral and fungal etiologies (e.g., herpes and tinea barbae), as well as keratosis pilaris, skin bumps resulting from the overproduction of keratin. Since folliculitis is a suppurative infection, warm compresses that allow a natural drainage of the detritus may be a sufficient treatment. A gentle cleansing of the skin and antibiotics active against Gram-positive flora may be helpful, as well [2].

### 4.4. Abscesses

An abscess, by definition, is a collection of pus within the body tissue. Furuncles (boils) or carbuncles (clusters of boils) are typically deeper than folliculitis, and more painful. If the affected area is easily accessible—and there is no overlying cellulitis—incision and drainage alone, performed as an office procedure, should be sufficient treatment [1,2,3]. Packing of larger abscesses such as infected pilonidal cysts is done to facilitate drainage of the pus, but not so tightly as to fill the potential space. Placing just a wick or drain may be preferable for children, as packing the wound is typically rather painful [14].

Other examples of abscesses frequently encountered in ambulatory facilities include dental abscesses, hidradenitis suppurativa, and pilonidal abscesses. Generally speaking, dental infections can involve the gums only (gingivitis and periodontitis) or the gums and teeth. Definitive treatment is rendered by a Dentist, but in the meanwhile, oral antibiotics such as amoxicillin, amoxicillin-clavulanate, clindamycin, metronidazole, and azithromycin may be needed. Periodontal infections often involve anaerobic bacteria.

Topical clindamycin or oral antibiotics such as doxycycline may be used for acute, painful inflammatory lesions from hidradenitis suppurativa. Despite the suppurative nature of this infection, incision and drainage should be reserved for severe cases, preferably under the guidance of a Dermatologist. Similarly, large or otherwise complicated abscesses (e.g., perianal abscesses) are often polymicrobial and may best be handled by a Colorectal Surgeon, as antibiotics alone may be insufficient. If it is necessary to drain a large abscess, making multiple counter-incisions is preferable to making a long, singular incision, which can delay wound healing and is more likely to cause deformity [1].

#### Recurrent Abscesses

If a patient suffers from recurrent abscesses, consider local causes such as hidradenitis suppurativa or an infected pilonidal cyst [14]. The treatment includes the use of warm compresses and antibiotics. Incision and drainage, with culture of the purulent material, may be necessary. If a patient has two or more infections at different body sites within a 6-month period, consider decolonization with an intranasal mupirocin ointment, since MRSA often resides in the nose [6]; chlorhexidine body wash [2]; and decontamination of personal items such as towels and bed linens. Oral medications are not routinely used for decolonization itself.

### 4.5. Cellulitis

Cellulitis is a deeper and poorly-demarcated SSTI that can invade lymph tissue and the blood. The treatment should be directed against typical Gram-positive pathogens—specifically streptococcus species. A 5-day course of antibiotics may actually be as effective as 10 days’ worth [3,13,14,25]. To avoid recurrence, tissue maceration, edema, eczema, and venous insufficiency should be treated, though this may be difficult, especially in an ambulatory context [2].

#### Recurrent Cellulitis

Similarly, for a patient with recurrent cellulitis, look for predisposing conditions. If the patient still experiences more than 3–4 episodes annually despite trying to control these factors, oral or parenteral antibiotic prophylaxis could be considered [2,14].

### 4.6. Erysipelas

Erysipelas is a more superficial form of cellulitis. It is a distinct entity from erysipeloid, which is associated with handling fish. It is colloquially known as “Saint Anthony’s fire” due to the intense, well-demarcated rash and burning sensation associated with it [2]. Erysipelas is more common in the extremes of age. It is typically caused by group A streptococci (GAS), but groups C and G streptococci have also been implicated [1]. Treatment for erysipelas and cellulitis includes antibiotics and potentially surgical intervention such as incision and drainage, surgical debridement, or even fasciotomy.

### 4.7. Necrotizing Infections

Since necrotizing infections often present a threat to life and limb, recognition of a necrotizing soft tissue infection (NSTI) is vital. When recognized early and treated promptly, the mortality rate for necrotizing fasciitis, for instance, drops from 23.5% to 10% [4]. SSTIs that seem to progress rapidly (i.e., within a couple of days), should be treated as a necrotizing infection until proven otherwise [1]. A history of penetrating or blunt trauma may also suggest a necrotizing infection. NSTIs can involve any and all layers of soft tissue, from the superficial dermis and subcutaneous tissue to deeper fascia and muscle, hence terms such as “necrotizing fasciitis” and “necrotizing myositis.” Regardless of the level of involvement, NSTIs are a medical emergency. They are classified by the bacterial pathogens present: Type 1 is polymicrobial (aerobic and anaerobic) and more likely in older or sick individuals; type 2 is monomicrobial and caused by GAS or MRSA. Type 3 is also monomicrobial and synonymous with gas gangrene (clostridial myonecrosis) [1,2]. Additionally, Panton-Valentine leukocidin (PVL), a cytotoxin produced by some strains of *S. aureus*, has been suggested to increase virulence, and should be considered in severe and/or necrotizing infections [26,27]. Necrotizing infections may also be named for the body part they affect—for example, Fournier’s gangrene, which occurs in the genital area and perineum but can easily spread to the abdominal wall, legs, and retroperitoneum.

Immediate surgical exploration is the only definitive means to diagnose a necrotizing infection [4]. En route, blood cultures should be drawn, but other laboratory tests are unlikely to offer much benefit. Though various imaging modalities may be helpful—plain radiographs excluded—they are often impractical in this context. Surgical source control should occur within the first 12 h of hospital admission. Indeed, mortality rates are higher in patients who have waited longer for surgical care. Fluid resuscitation—there is no “ideal” fluid currently—and broad-spectrum IV antibiotics are given and the wound is left open. Amputation is a last resort [1].

### 4.8. Special Situations

SSTIs in injection drug-users (IVDUs) tend to be polymicrobial, so consider combination therapies (e.g., cephalexin and metronidazole) [5]. Diabetics are particularly susceptible to wounds on their feet. As such, they should be checking their feet daily and having regular exams with their primary care provider (PCP), Endocrinologist, and/or Podiatrist. Diabetic foot infections (DFIs) may be more extensive than they appear and are usually not painful to the patient due to peripheral neuropathy [2,5]. Apparent SSTIs in immunocompromised individuals come with a litany of other possible etiologies for the signs and symptoms, including bacterial, viral, fungal, parasitic, and autoimmune causes [14]. A biopsy may be helpful.

#### 4.8.1. Bite Wounds

Animal—and sometimes human—bites are another common presenting concern in walk-in clinics and facilities. Who (or what) did the biting? Post-exposure prophylaxis (PEP) against rabies is indicated after mammalian bites or scratches in most countries. Report the bite to animal control and consult with local health officials [14]. Human bites that have broken the skin and drawn blood—even if unintentional, as from a fight—can theoretically transmit hepatitis B, C, and HIV [28]. A patient who has sustained a bite (or any other puncture wound, of course) should confirm that their tetanus booster is up to date. Unfortunately, signs of infection from a bite may have a delayed presentation, 24–72 h after injury. Overall, between 10–20% of bite wounds become infected. As many as 30–50% of cat bites will [1]. Human bites often occur in the context of a fight (a so-called “fight bite”): The pugilist’s fist strikes the other person’s teeth, resulting in a penetrating injury to the extensor tendon and metacarpophalangeal (MP) joint capsule [29]. By comparison, cat bites are penetrating and affect the deep tissue and dog bites are more tearing and destroy the tissue. Prophylaxis with antibiotics is not universally recommended [1]. There is a weak evidence, but experts do recommend early antibiotics for fresh, deep wounds and wounds in so-called critical body areas—for instance, the hands, feet, face, genitals, and near the joints [1].

Other risk factors that would suggest the early use of prophylactic antibiotics include immunocompromise, advanced liver disease, and preexisting or resultant edema of the affected area. Cover for aerobic and anaerobic bacteria. Amoxicillin-clavulanate, ceftriaxone and metronidazole, or trimethoprim-sulfamethoxazole and clindamycin are good choices. Gentle irrigation of the bite wound can help remove foreign debris and pathogens. Primary wound closure is not recommended, except for wounds on the face [1,2,14].

#### 4.8.2. Other SSTIs and Mimics

Non-infectious etiologies—such as thrombophlebitis, deep venous thrombosis, contact dermatitis, and gout—can mimic SSTIs, as they may also present with erythema, warmth, and tenderness [4,12,13,30]. Ingrown nails—most commonly on the great toe—can look red and moist but are not always infected. Paronychia, a purulent infection in the nail gutter, is more likely in the fingers, potentially due to seeding with oral flora when someone bites their nails. Infection of the pulp or the pad of the finger, unceremoniously called a “felon,” is also due to bacteria. A herpetic whitlow, by contrast, is due to herpes simplex virus, though in milder presentations, it can look a bit as dyshidrotic eczema. Other viral skin infections elsewhere on the body include shingles, herpes gladiatorum, herpes labialis, and herpes genitalis. Pyogenic flexor tenosynovitis, a deep hand infection that most likely results from penetrating trauma, may be secondary to bacterial, viral, or fungal pathogens [29]. Pyogenic granulomas, on the other hand, which can occur on the digits, the lips or gums, or really anywhere on the body, are reactive malformations of capillary blood vessels. They are benign but bleed profusely. Due to their “raw” appearance—just like ingrown toenails or granulation tissue—sometimes pyogenic granulomas are mistaken for an infection.

## 5. Conclusions

Since the entities comprising the general concept of SSTIs are diverse, consider the patient’s health at the baseline and if they appear ill on presentation, where the infection is (and how deep), if it is suppurative or not, and if there is evidence of tissue necrosis. The diagnosis of SSTIs is clinical, which is often pattern recognition. Treatment decisions are based on these factors but subject to revision if the patient does not appear to be on the path towards convalescence [1,3,5].

## Figures and Tables

**Table 1:** Risk factors for skin and soft tissue infections (SSTIs).

General Risk Factors	Risk Factors for MRSA Infections
Cardiopulmonary disease	Younger age
Hepatorenal disease	Health care professionals
Older age	Military personnel
Debility	Dialysis
Obesity	Long-term intravascular access
Asplenia	Prolonged hospitalization
Immunocompromise (e.g., HIV, chemotherapy)	
Peripheral arteriovenous insufficiency	**Risk factors for NF**
Peripheral neuropathy	Alcohol abuse
Lymphedema	Poor nutrition
Water exposure (salt or freshwater)	Sports participation
Human/animal bites	Trauma
Intravenous or subcutaneous drug use	Surgery

**Table 2:** Simplified management of SSTIs (adapted from [14]).

	Purulent	Non-Purulent
**Mild**	Incision and drainage (I & D)	Topical antibiotics Oral antibiotics *(cover GAS)*
**Moderate/severe**	I & D Antibiotics *(cover MRSA)* Surgical debridement	Parenteral antibiotics Surgical debridement

## Data Availability

No new data were created or analyzed in this study. Data sharing is not applicable to this article.
